# Phytosterols and γ-Oryzanol as Cholesterol Solid Phase Modifiers during Digestion

**DOI:** 10.3390/foods11223629

**Published:** 2022-11-14

**Authors:** Eduardo S. Esperança, Mariane S. Bonatto, Karen C. G. Silva, Gustavo G. Shimamoto, Matthieu Tubino, Mariana C. Costa, Christianne E. C. Rodrigues, Antonio J. A. Meirelles, Ana C. K. Sato, Guilherme J. Maximo

**Affiliations:** 1 School of Food Engineering, University of Campinas, Campinas 13083-862, Brazil; 2 Chemical Institute, University of Campinas, Campinas 13083-970, Brazil; 3 School of Chemical Engineering, University of Campinas, Campinas 13083-852, Brazil; 4 Faculty of Animal Science and Food Engineering, University of São Paulo, Pirassununga 13635-900, Brazil

**Keywords:** solid–liquid phase equilibrium, liquid crystal, cocrystal, solid solution

## Abstract

Literature reports that ingestion of phytosterols and γ-oryzanol contributes to cholesterol lowering. Despite in vivo observations, thermodynamic phase equilibria could explain phenomena occurring during digestion leading to such effects. To advance the observations made by previous literature, this study was aimed at describing the complete solid–liquid phase equilibrium diagrams of cholesterol + phytosterol and γ-oryzanol systems by DSC, evaluating them by powder X-ray, microscopy, and thermodynamic modeling. Additionally, this study evaluated the phenomena observed by an in vitro digestibility method. Results confirmed the formation of solid solution in the cholesterol + phytosterols system at any concentration and that cholesterol + γ-oryzanol mixtures formed stable liquid crystalline phases with a significant melting temperature depression. The in vitro protocol supported the idea that the same phenomena can occur during digestion in which mechanochemical forces were probably the mechanisms promoting cholesterol solid phase changes in the presence of such phytocompounds. In this case, these changes could alter cholesterol solubility and possibly its absorption in the gastrointestinal lumen.

## 1. Introduction

**Figure 1:**
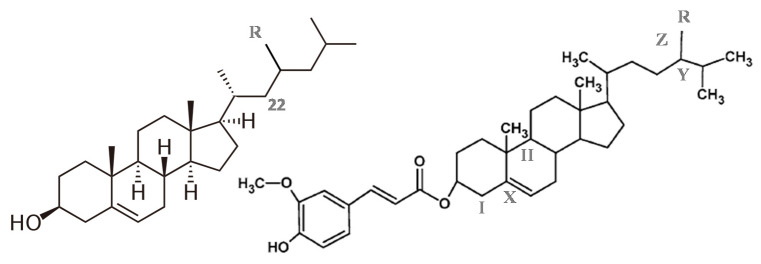
Top: at the right, the structure for cholesterol (R = H), β-sitosterol (R = CH_2_CH_3_), stigmasterol (R = CH_2_CH_3_, double bond at C22), and campesterol (R = CH_3_), adapted from literature [7]; at the left, principal structure for γ-oryzanol main ferulates: campesteryl ferulate (R = CH_3_); and sitosteryl ferulate (R = CH_2_CH_3_); cycloartanyl ferulate (X = single bond, I = 2 CH_3,_ II = Ciclopropane, R = (-)); Cycloartenyl ferulate (the same with Y = double bond); 24-metilene cycloartanyl ferulate (the same with R = CH_2_, Z = double bond); adapted from Cuevas et al. [8].

Among the biodrugs acting as reducers of cholesterol levels in the human body, phytosterols have been certainly one of the most studied in the literature. Phytosterols are sterols, as well as cholesterol, especially found in the composition of vegetable oils, such as canola, olive, corn, palm, and soybean [1]. There are more than 40 types of sterols of different plants identified, but the most abundant is β-sitosterol, although it is generally found in mixtures composed of 70% β-sitosterol and 30% of β-sitostanol and campesterol (Figure 1) [2]. Their intake has been directly associated with the decrease of the blood plasma cholesterol as well as the molecular lipoprotein LDL complex, through a cycle that, according to several authors, starts with the inhibition of the cholesterol absorption from the diet in the intestine [2,3,4]. As a result, less cholesterol is transported into the enterocytes being excreted in the feces [5,6]. Consequently, for the maintenance of cholesterol homeostasis in the organism, the metabolism that rules the cholesterol accumulation in the liver as well as the formation of the lipoprotein LDL is altered, promoting its reduction in the blood plasma.

Another phytocompound that deserves special attention in this context is γ-oryzanol. This biocompound is in fact a multicomponent complex of ferulic esters, sterols, triterpenic alcohols, tocopherols and tocotrienols [9,10] which has been also reported, through in vivo studies, as a hypocholesterolemic agent [7,11,12]. This is particularly interesting considering its predominant food matrix, rice bran oil, widely consumed in East Asian countries, but also in Western countries, especially due to such a nutraceutical potential [13].

Several Health organizations around the world [14,15,16,17,18] officially recommend the daily intake of phytosterols in addition to a healthy diet to ensure the reduction of blood cholesterol, considering that hipocholesterolemia has no chronic origins. As a lipophilic biocompound, phytosterols are easily soluble in lipidic matrices [19,20] and, for this reason, their addition in margarine, spreads or yogurts is highly applied [5,21].

The mechanisms of blood cholesterol lowering through the ingestion of phytosterols are not a recent issue and some theories are already the scope of several studies [2,19,22] being the “cocrystallization” mechanism the most diffused [23,24,25]. Through this mechanism phytosterols and cholesterol could form a “cocrystal”, also called a “solid solution”, in a stricter thermodynamic definition, whose absorption is probably lower than the single crystal of cholesterol [26,27]. The cocrystallization is a process largely used in the pharmaceutical industry for the development of crystalline structures for the transport of drugs and controlled release in the body, as it alters the stability, solubility, and bioavailability of the biomolecule carried, while maintaining its structure intact [28]. Cocrystals also naturally occur in systems rich in lipidic compounds [29,30,31], resulting in different thermal and mechanical properties [32,33,34,35].

Although cocrystallization is a clue to explain the mechanism of cholesterol-lowering with the ingestion of phytosterols, studies in the literature are still scarce. Information on the lowering mechanisms with the ingestion of other compounds is also scarce, which is the case of γ-oryzanol, as well as the systematization of the phase equilibrium effect of such biocompounds mixed with cholesterol. This means that despite mixtures of cholesterol and such phytochemicals have been previously evaluated in literature by in vivo studies and other approaches, the description of the complete solid–liquid equilibrium phase diagrams, including their thermodynamic evaluation, could reveal effects unknown and how temperature and concentration could affect the phenomena observed have not been yet explored. In addition, as far as we know no in vitro protocol was employed to verify the phenomena observed. In this context, this work was aimed at determining the complete solid–liquid equilibrium behavior of the pseudo-binary systems cholesterol + phytosterol and cholesterol + γ-oryzanol to increase the comprehension of the phenomena possibly occurring during digestion. This work additionally verifies if the phase behavior observed is reproduced in the gastrointestinal system by using a complete in vitro digestibility protocol [36].

## 2. Materials and Methods

### 2.1. Phase Equilibrium Behaviour

The formulation of the pseudo-binary systems (1 g, approximately, at each concentration), Cholesterol (Sigma-Aldrich, Saint Louis, MO, USA, purity of 95% w/w, analyzed in DSC software, Pyris Analyzer v.11, Waltham, MA, USA) a mixture of phytosterols composed of b-sitosterol (>70% w/w), b-sitostanol and campesterol (Sigma-Aldrich), and γ-oryzanol (Tsuno Rice Fine Chemicals, Wakayama, Japan, 99% w/w purity, a composition described by Cuevas et al. [37] with a molar mass of 605.32 g/mol, calculated by its composition) were used. Systems were formulated in molar fractions between 0.0 and 1.0 (9 mixtures + two pure conditions) in an analytical balance (Precisa Gravimetrics AG, Dietkon, Switzerland, precision of 2 × 10^−4^ g). Samples were magnetically stirred at a temperature higher than the melting of the samples (T > 140 °C), under an inert atmosphere (Nitrogen > 99.9% w/w, White Martins, Danbury, CT, USA), and sequentially stored at a temperature lower than 273 K.

Differential Scanning Calorimetry (DSC) was applied by using a DSC8500 calorimeter (PerkinElmer, Waltham, MA, USA) for the determination of the mixtures’ phase equilibrium profiles. Using a micro-analytical balance AD6 (PerkinElmer, Waltham, MA, USA, precision of 2 × 10^−6^ g), samples of up to 5 mg were gravimetrically prepared and sealed in aluminum pans. For the phytosterol system, a heating-cooling cycle was used at 1 K·min^−1^, following methodologies previously described in the literature [38,39,40,41]. For the γ-oryzanol system the same cycle was applied at 5 K·min^−1^. After previous tests, it was verified that lower rates prevent the γ-oryzanol transition resolution. This was assumed to be due to its liquid crystalline behavior, further explained in the results section. The Perkin Elmer analysis software (Pyris Analyzer v.11, Waltham, MA, USA) was used for evaluating the transition temperatures, established as the peak top temperatures due to the appearance of extended peaks. Additional details on the melting behavior of mixtures were obtained by optical polarized light microscopy (DM2500, Leica Microsystems GmbH, Wetzlar, Germany) equipped with a temperature control stage (LTS420, Linkam Scientific Instruments Ltd., Tadworth, UK). A heating procedure was applied, similar to what was used for DSC [30,38,39].

The crystalline structures of the mixtures (formation of cocrystal and other structures) were evaluated by powder X-ray diffractometry (Philips X’Pert, Philips Analytical, Almelo, Netherlands). The method used an anode reflection mode Cu–K (λ = 1.5406 Å) and graphite monochromator, with the obtaining of diffractograms from 0° to 200° with a Bragg-Brentano geometry (θ:2θ), and steps of 0.02° each 2s [29,40,41].

The experimental SLE data were used for the elaboration of an SLE phase diagram, representing the transition temperatures as a function of the concentration of the system, expressing the regions in which the system comprises liquid, solid and liquid crystalline phases. The systems’ melting behavior was modeled by the SLE Equation [42] (Equation (1)).

(1) $$\ln\frac{x_{i}\gamma_{i}^{L}}{z_{i}\gamma_{i}^{S}}=\frac{\Delta_{fus}H}{R}\left(\frac{1}{T_{fus}}-\frac{1}{T}\right)$$

where *x*, *z*, and g are the mole fraction of the components in liquid (*L*) and solid (*S*) phases, and the activity coefficients of the compounds. *R* is the gas constant (8.314 J∙mol^−1^∙K^−1^), *T* is the mixture melting temperature (K), Δ*_fus_H* and *T_fus_* stand for the melting enthalpy (J/mol) and temperature (K) for each compound. The 3-suffix Margules equation [43] was used for the calculation of the activity coefficients (γ). The resolution of Equation (1) and the adjustment of the parameters of the Margules equation (parameters *A* and *B*, for each phase, indicated in results) were done by using the *Cristal-T* algorithm, described elsewhere [30].

### 2.2. In Vitro Digestibility

Cholesterol + phytosterol system at *x* = 0.5 cholesterol molar fraction and cholesterol + γ-oryzanol system at *x* = 0.6 cholesterol molar fraction were each one mixed with water at a mass ratio of 1:5 (system:water) and subjected to the in vitro digestibility analysis. Choices for molar fractions are based on experimental results and will be further explained. Pure cholesterol was also submitted to the analysis as standard behavior. The methodology applied was the one proposed by [36]. The method is a static-based protocol with 3 steps. The first step simulates the oral phase that, in this case, was neglected due to the absence of carbohydrates (according to the protocol). The second and third steps simulate the gastric and enteric phases. The protocol started adding the pure compounds at room temperature (25 °C), and water to the vessels without previous treatment, simulating the ingestion in a diet. The system was conditioned in a jacketed system and submitted to magnetic stirring at a low rate. The temperature was controlled by a thermostatic bath set to 37 °C. The gastric digestion was simulated by adding a Simulated Gastric Fluid (SGF), porcine pepsin at 25,000 U.mL^−1^, 0.3 M CaCl_2_ and 1 M HCl for adjustment of the pH to 3,0. The pH was controlled by a pHmeter, and the system was stirred for 120 min. The enteric digestion was sequentially simulated by adding a Simulated Intestinal Fluid (SIF), porcine bile, pancreatin 800 U.mL^−1^, 0.3 M CaCl_2_ and 1 M NaOH for adjustment of the pH to 7.0. The pH was controlled by a pHmeter, and the system was further stirred for 120 min. Amounts of simulated fluids, CaCl_2_ and enzymes are found in that work. Two processes were simulated in parallel. At the end of the gastric step, one simulation was stopped, and the solid phase was collected, filtered under vacuum, and immediately cooled in an ultrafreezer (193 K) to stop the enzymatic action. At the end of the second step, the remained simulation was stopped, followed by the same procedure. The solid phases after gastric and after the enteric step were analyzed by polarized optical microscopy (POM) and DSC.

## 3. Results and Discussion

### 3.1. Phytosterol and γ-Oryzanol Melting Behaviour

**Figure 2:**
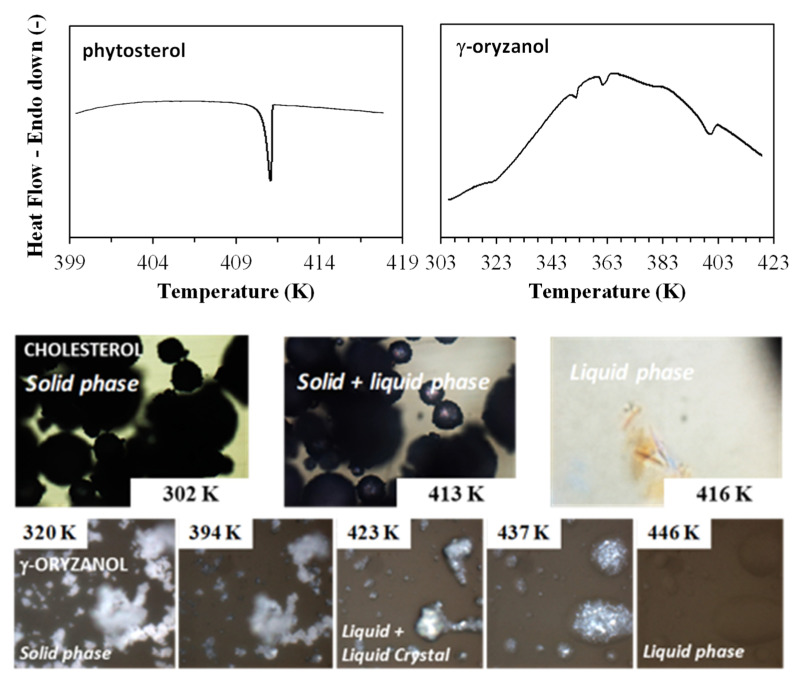
DSC thermograms for phytosterol and γ-oryzanol melting process and images obtained by POM for phytosterol and γ-oryzanol melting process.

The cholesterol melting profile is that presented in previous works [44]. Figure 2 shows the thermogram for the sample of phytosterol. The melting profile showed a very well-defined thermal event at a peak temperature of 411.16 ± 0.24 K, very similar to the cholesterol melting profile. The melting enthalpy found for this pseudo-biocompound was 20.4 ± 0.26 kJ/mol. No value for melting temperature or enthalpy was found in the literature for such phytosterols (considering the National Institute of Standards and Technology, NIST, databank). There are more than 40 types of phytosterols, being β-sitosterol the most abundant [2]. Considering that a commercial mixture of phytosterols was used in this work one should expect that more than 1 peak was observed in the DSC thermogram. However, if only one peak was seen, such a mixture of phytosterols probably formed a solid solution (cocrystal). In fact, this behavior is corroborated by the very similar phytosterols’ molecular structures (Figure 1). During crystallization, the peak is very sharp and well-defined. It means that, due to the complexity of the structure, when enough energy is removed from the system the mobility of the molecule is drastically altered, favoring the packing of the structure and its crystallization.

The melting behavior of the phytosterol sample was also analyzed by polarizing optical microscopy (POM) and presented in Figure 2. POM showed a “slightly broad” melting profile, in agreement with the DSC data. It means that the initial melting of this pseudo-biocompound was a few degrees lower than the peak temperature observed in DSC and finished few degrees higher than that. This is probably due to the complexity of the molecule that avoids a sharp and well-defined melting process, like what occurs to cholesterol, which is also a sterol [44].

Figure 2 also shows the thermogram for the γ-oryzanol sample. Thermograms were firstly obtained at 1 K/min, which according to the literature [45] simulates a situation of a quasi-equilibrium state, ideal for the description of the compounds’ melting behavior. However, no peaks were seen. A higher rate of 5 K/min was then used, providing a better resolution. The thermogram indicates several thermal transitions between 323.35 and 411.16 K, which was expected considering that γ-oryzanol is a mix of compounds. POM was also used for evaluating this complex profile (Figure 2). Micrographs showed a melting behavior with wide temperatures. Moreover, during the samples’ melting, the polarized light could show the formation of a liquid crystalline phase, characterized by a pattern such as what the literature refers to as a “marble texture”. Such a pattern reveals the formation of a lamellar liquid crystalline structure, which was also observed in literature for this pseudo-compound [46]. At temperatures higher than 392 K (last DSC transitions) a liquid-crystalline + isotropic liquid region (black region) occurred. The disappearance of the liquid crystalline phase can be observed by increasing of the dark background in the micrographs up to 439 K. The literature affirms that, due to the appearance of a liquid crystalline structure at a large temperature range, γ-oryzanol is an interesting gelator in organic systems [46].

### 3.2. Cholesterol + Phytosterol Solid–Liquid Equilibrium Phase Diagram

Figure S1 (Supplementary Material) presents the DSC thermograms obtained for the cholesterol + phytosterol system. They show a single thermal event for all compositions and at a temperature slightly lower than the melting temperatures of the pure compounds. The melting temperatures followed the tendency in which a minimum point is achieved between *x* = 0.45 and *x* = 0.50 cholesterol molar fraction. Classically, when two compounds are mixed in the liquid phase, during the recrystallization, the compounds are crystallized independently. The melting temperature of the system is decreased establishing a minimum point called the eutectic point. If so, the DSC should present two events, the melting of the eutectic composition, and the melting of the mixture.

Since, in this case, no eutectic transition was observed, one could assume that during crystallization, compounds formed a unique crystal structure, what the literature calls a solid solution (or cocrystal) [47,48]. To form a solid solution, it is necessary that the crystals of the molecules involved in the process have similar crystalline structures [26,27] or that, during the crystallization of the system, one of the compounds is conformed to the crystalline structure of the other compound. The literature has already indicated the formation of cocrystals between cholesterol and phytosterols [20], but no formal solid–liquid phase equilibrium diagram.

**Figure 3:**
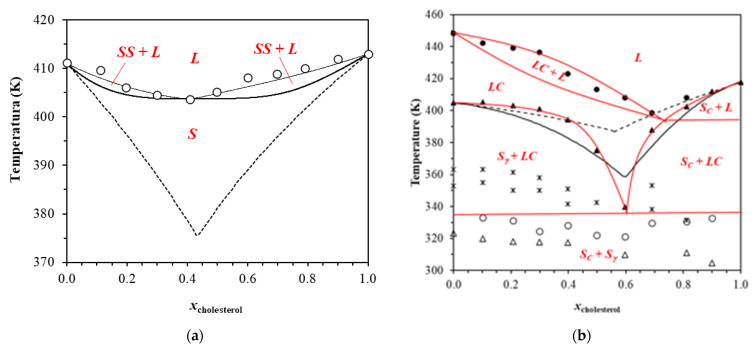
(**a**) SLE diagram for cholesterol + phytosterol; DSC peak top melting temperatures (○); Equation (1) (continuous lines, solid phase parameters *A* = 6.5 kJ/mol; *B* = −0.3 kJ/mol; liquid phase parameters *A* = 4.5 kJ/mol; *B* = −0.1 kJ/mol); ideal behavior (dashed lines). (**b**) SLE phase diagram for cholesterol + γ-oryzanol: 1st transitions for DSC (△) and POM (○), DSC solid transitions (∗), DSC melting temperatures (▲), Liquid crystal disappearance (●). Dashed lines are the ideal behavior; Black lines are Equation (1) considering a simple eutectic profile (Liquid phase parameters *A* = −13.1 kJ/mol; *B* = −1.9 kJ/mol); Red lines are the possible interpretation of the diagram. *L* = Liquid Phase; *LC* = Liquid Crystal; *S_C_* = cholesterol solid phase and S_g_ = γ-oryzanol solid phase.

Figure 3 shows the complete solid–liquid Equilibrium phase diagram for this mixture, containing the experimental data obtained by DSC and POM for each mixture (from 0 to 1 molar fraction of cholesterol). Table 1 provides the complete experimental data. The phase diagram also shows two theoretical curves calculated by using Equation (1). Dashed lines show the real behavior, adjusted by the activity coefficient equation, and the continuous curve shows an ideal behavior, supposing that the mixture behaves as a eutectic mixture. For this, Equation (1) was modified such that the γ_i_^L^ = 1 and *z*_i_.γ_i_^S^ = 1 (no solid solution is formed).

Results show that this system is far from the ideal behavior. In fact, the values obtained for the activity coefficients of the compounds in the solid phase are very close to 1. This result corroborates with the solid solution formation. The SLE profile proposed here presents 4 regions: 1 solid phase composed of a cocrystal formed between these 2 compounds, 2 biphasic regions formed by the cocrystal and the liquid phase, and 1 liquid phase. The biphasic region at the right-hand side of the diagram is composed of a solid solution rich in cholesterol and at the left-hand side, by a solid solution rich in phytosterol. This profile is similar to the azeotropic behavior observed in case of the vapor–liquid equilibrium theory. This system was further evaluated by thermal controlled polarized optical microscopy and powder X-ray diffraction.

**Figure 4:**
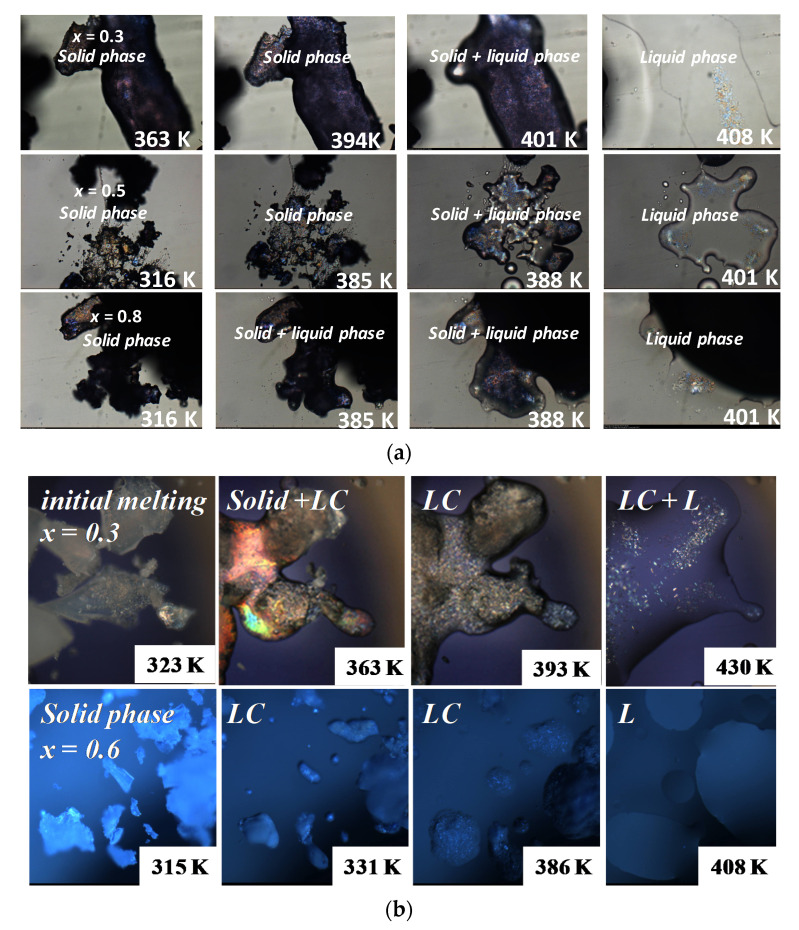
(**a**) Micrographs for cholesterol + phytosterol mixtures and for (**b**) cholesterol + γ-oryzanol mixtures (LC = Liquid Crystal; L = Liquid). *x* = cholesterol molar composition.

Figure 4 shows some thermomicrographs at different temperatures and concentrations. A narrow biphasic region appeared, corroborating the theoretical curve. The initial melting process occurred at different temperatures and was very close to the final melting temperatures, corroborating the absence of the eutectic point. Indeed, classically, lipidic systems composed of fatty acids, fatty alcohols or triacylglycerols usually present a significant melting temperature depression, with indicates the presence of a eutectic transition [38,39,40,41,45], which is not the case for this system. Moreover, in the case of a eutectic system, above the eutectic temperature, solid–liquid regions are observed throughout the concentration range [38,39,40,41,45], which is also not the case in this mixture. Microscopies also show that the crystalline behavior of the solid phase at the right-hand side of the diagram (Figure 3, x = 0.3) was slightly different from those at the left-hand side (Figure 3, x = 0.8). This is due to the solid solutions formed: at one side rich in cholesterol and on another side rich in phytosterol. Different solid phases were also observed in some works in literature when solid solutions are formed, even in a small region of the diagram [30]. This profile corroborates the SLE phase diagram proposed.

**Figure 5:**
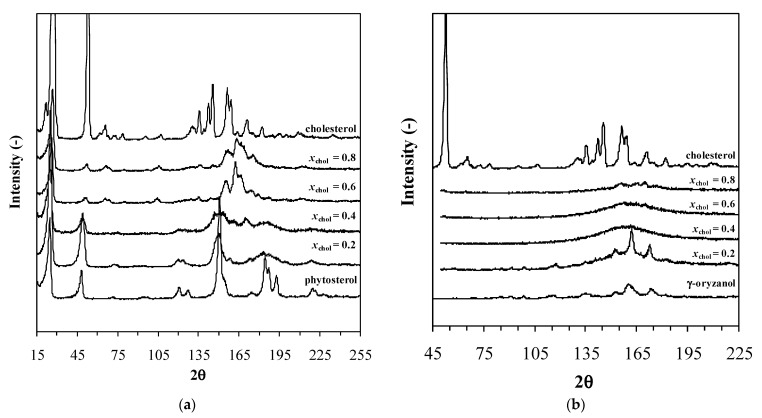
Powder X-ray difractograms for (**a**) cholesterol + phytosterol and (**b**) cholesterol + γ-oryzanol mixtures.

Figure 5 shows the powder X-ray diffractograms. Pure cholesterol and phytosterol presented different diffractometric patterns. For mixtures, other patterns were observed. This indicates that mixtures present different crystalline structures when compared to pure compounds, which is a characteristic of the formation of a solid solution. If a solid solution is not formed, mixtures would present a summation of the formers’ patterns, i.e., the same peaks at the same angles. Indeed, according to Ref. [49], a solid solution is a thermodynamic situation in which a compound is fitted to the crystal lattice of other compounds, forming a unique crystalline cell. This crystalline cell can be similar to one or other former compound, which was observed by Maximo and co-workers in works where lipidic systems were evaluated [29,30]. In this work, for concentrations closer to pure cholesterol (x = 0.6 and 0.8, Figure 4) mixtures presented some peaks at the same angles observed for pure cholesterol, meaning that, in this range, cholesterol acted as the “host” of the crystalline unit cell of the solid phase. Otherwise, for concentrations closer to phytosterol (x = 0.2 and 0.4, Figure 4), diffractograms presented some peaks at the same angles that were observed for phytosterol. This means that, in this region, the cocrystal (solid solution) adopted the crystalline matrix of the phytosterol, and in this case, this pseudo-compound acted as the “host” of the structure.

### 3.3. Cholesterol + γ-Oryzanol Solid–Liquid Equilibrium Phase Diagram

Figure S2 (Supplementary Material) shows the thermograms obtained for the cholesterol + γ-oryzanol system, Figure 3 shows the interpretation of the possible SLE thermodynamic profile, based on data analysis, presented in Table 1, and Figure 4 shows some micrographs. The phase diagram also shows two theoretical curves calculated by using Equation (1). Dashed lines show the ideal behavior and black lines consider a eutectic system, i.e., *z*_i_.γ_i_^S^ = 1 (no solid solution is formed). The red lines show a probably real interpretation. At each concentration, both DSC and microscopy show several thermal transitions. Some of them (T_tr2_ and T_tr3_, Table 1) were attributed to solid phase transitions of γ-oryzanol. According to POM initial melting of mixtures occurred, approximately, at the same temperature, around 330 K (Figure 3, ○), which is a typical profile of a eutectic behavior. The eutectic behavior could be also observed in the profile established by the melting temperature observed in DSC: the temperature decreased up to a minimum point, close to x = 0.6 when the temperature started to increase (Figure 3, ▲).

Figure 3 shows the mixture’s melting at *x* = 0.3 and *x* = 0.6. At *x* = 0.3, after the initial melting a concentrated and stable liquid-crystalline mesophase was clearly observed together with a solid phase. The mesophase could be verified until high temperatures. The sample at *x* = 0.6 clearly melts close to 330 K with no remained solid phase. These results confirmed the eutectic profile observed with a minimum point close to *x* = 0.6.

Notedly, together with the formation of a thermal stable liquid-crystalline mesophase, a significant solid phase melting depression was observed in this system, with a eutectic point close to *x* = 0.6 cholesterol molar fraction. The ideal thermodynamic curve (Figure 3, dashed lines) is, in fact, clearly far from the real phase behavior. The possible interpretation of the SLE profile (following the Gibbs phase rules) proposes 7 regions: 1 solid phase composed of both compounds, 2 biphasic regions formed by liquid crystalline mesophases and solid compounds, 1 liquid crystalline mesophase, 2 biphasic regions with the liquid crystalline mesophase, one with the liquid phase and one with solid pure cholesterol, and 1 liquid phase. The γ-oryzanol proved to cause a significant altering in the cholesterol solid phase crystalline habit and, therefore, in its melting behavior. The liquid-crystalline (*LC*) mesophase formation and the high melting depression are probably related to the beneficial effects associated to the use of this bioactive, claimed as a hypocholesterolemic compound.

The powder X-ray analysis corroborates with these assumptions (Figure 5). The mixtures’ diffractograms were clearly dispersed with poor defined peaks. This occurred even though analysis occurring at 298 K, i.e., at the solid phase. One might suggest that due to the formation of the liquid-crystalline mesophases, during crystallization, a significant fraction of the solid phase was not crystallized, keeping amorphous. This is quite significant and suggests that the cholesterol solid phase is, in fact, largely affected in the presence of γ-oryzanol.

### 3.4. Phase Behavior of the Mixtures after In Vitro Digestibility

**Figure 6:**
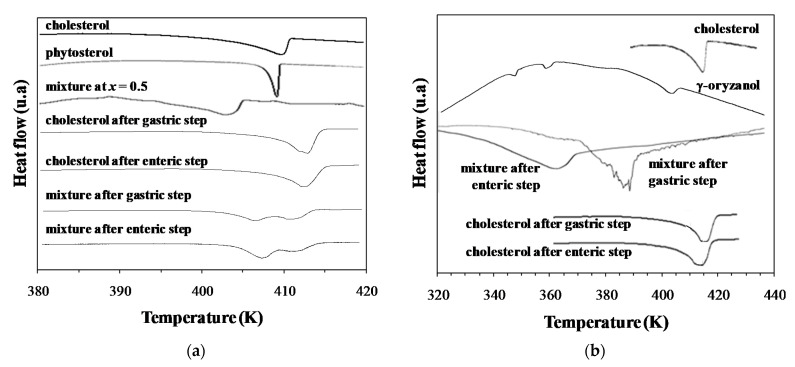
DSC thermograms for cholesterol and their mixture with (**a**) phytosterol and (**b**) γ-oryzanol before and after digestion.

Considering that at any concentration cholesterol + phytosterol formed solid solutions and, in case of cholesterol + γ-oryzanol the minimum temperature occurred at *x* = 0.6 cholesterol molar fraction. Two “model meals” composed of cholesterol + phytosterol at *x* = 0.5, and cholesterol + γ-oryzanol at *x* = 0.6 were submitted to the Minekus et al. [36] in vitro digestibility protocol. Pure compounds were put in the simulated digestive system, both at the solid phase, without any pre-mix (i.e., unlike the study of solid-liquid equilibrium, where the samples were mixed and melted before the experiment). This could simulate the ingestion of such phytocompounds in a food matrix and their interaction with endogenous and food cholesterol. Pure cholesterol was also submitted to digestion for comparison purposes. Figure 6 shows the DSC thermograms of the solid phases before and after the gastric and enteric steps of the simulated digestions.

**Figure 7:**
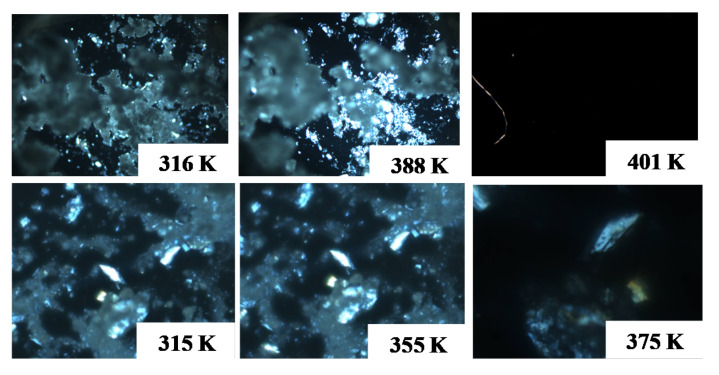
Micrographs at different temperature for cholesterol + phytosterol (**1st row**) and cholesterol + γ-oryzanol (**2nd row**) after digestion.

The cholesterol + phytosterol system at *x* = 0.5 presents a melting temperature close to 403 K, lower than pure compounds, with melting temperatures higher than 413 K. The DSC thermograms of the solid phase of the cholesterol sample during digestion presented one thermal event with a melting temperature close to the cholesterol melting temperature. It means that probably nothing happened to the crystalline structure of cholesterol during digestion. Otherwise, the DSC thermograms of the solid phase of the mixture during digestion showed two thermal events, one close to 405 K, and another close to 413 K. The first peak is probably related to the melting temperature of the cocrystal formed by the mixture and the second is the overlapped melting temperature peaks of both pure compounds, whose melting points are very similar. Since these two peaks were observed, a fraction of pure compounds probably remained. The DSC thermograms obtained after the enteric step show that the 1st peak was more intense, probably indicating that the amount of cocrystal increased. Figure 7 shows some micrographs obtained in this essay. The melting temperature range of the solid phase obtained after digestion was close to those observed for the mixture prepared previously, which could really indicate the formation of the cocrystal. Moreover, the melting temperature was cleared and decreased compared to pure compounds.

In the case of the cholesterol + γ-oryzanol mixtures, DSC thermograms showed that a clear modification occurred in the solid phase after digestion. For the solid sample after the gastric step, the last thermal transition was observed close to 385 K, much lower than for pure cholesterol or γ-oryzanol melting temperatures. The same occurred after the enteric step, whose last thermal transition occurred close to 365 K. That is, there was a significant decrease in the melting point of cholesterol in the gastric system with the addition of γ-oryzanol. The same profile was observed in the thermal microscopies of the solid phase of this mixture after digestion where at 375 K the mesophase was almost melted.

In the first part of the work, the cocrystal (solid solution) in the case of the phytosterol system, and the melting temperature depression in case of γ-oryzanol, occurred after melting and recrystallization. This promoted molecular interaction, favoring the occurrence of the observed phenomena. However, no melting occurs in the digestion which indicates that other phenomena would be the clue for such effects. Mechanochemistry or mechanosynthesis is a term used in literature in which mechanical forces, such as those promoted by grinding in mills, induced the reaction/interaction, normally of solids. This is also a procedure used for the formation/formulation of solid solutions in mechanical, chemical, and pharmaceutical industries [50,51] by using several routes. Specific procedures such as “liquid-assisted grindings” or “ion- and liquid-assisted grinding”, for example, are mechanochemical reactions used in order to enhance the molecular mobility in a reactor with a solid-state reaction for the promotion of the formation of cocrystals [52]. This phenomenon can easily explain what probably occurs during digestion. In this case, also a solid-state reaction occurs in a presence of a liquid and a strong ionic media composed of salts, acids, and bases. This situation, under the action of mechanical forces of the peristaltic movements of the digestive system, can probably promote the interaction between the solid compounds (phytosterol/γ-oryzanol and cholesterol) through the formation of strong hydrogen-bonded links and, therefore, induce the formation of a unique crystal to unite cell, i.e., the solid solution, in case of phytosterol and a liquid-crystal state, in case of γ-oryzanol. One might observe that the in vitro simulation could not reach the intensity and the real mechanical forces established by the peristaltic movements that, in this case, were mimicked by magnetic stirring [53]. Therefore, due to the limitations of the in vitro model used, a remained fraction of unreacted solid compounds was observed.

## 4. Conclusions

Experimental and theoretical results here obtained showed that phytosterols and γ-oryzanol can promote significant modifications in the thermodynamic phase behavior of cholesterol. Mixtures of phytosterols and cholesterol formed solid solutions (cocrystals) at any concentration: the melting temperature of the mixtures, in this case, was closer to the melting temperature of the former compounds (between 403 and 410 K, approximately). The same behavior could be observed during simulated digestion, showing that the mechanochemical process was probably able to produce solid solutions when phytosterol and cholesterol are mixed. The γ-oryzanol could produce thermal-stable liquid crystalline structures in mixtures with cholesterol, also throughout the concentration range studied. Moreover, the melting temperature of the mixtures, in this case, was also decreased in almost 60 K at the molar ratio close to 60:40 when compared to the melting temperatures of the former compounds. Formation of liquid crystals and melting temperature depression could be also observed after simulated digestion.

These results proposes that when these nutraceutical compounds are ingested during a meal or as supplements, they could possibly interact with cholesterol (obtained from diet, for example) in the gastrointestinal lumen (during digestion) forming stable solid solutions (in case of phytosterols) or liquid crystals (in case of γ-oryzanol). In this case, results also showed that such modifications are promoted at any biocompound:cholesterol mixture ratio. When compared to pure cholesterol, these new phases formed (solid solutions or liquid crystals), by promoting cholesterol solid phase thermodynamic modifications, could modify cholesterol solubility in the aqueous gastrointestinal systems, probably modifying (even preventing) its absorption in the gastrointestinal tract. This idea corroborates to the hypocholesterolemic effects attributed to phytosterols and γ-oryzanol, observed in literature by using in vivo studies, as well as to the recommendations of World Health organizations for ingestion of these phytochemicals. Results also highlight the importance of introducing these important nutraceuticals on daily diet through *in nature* food or as ingredients, addictive, or coadjutants in food products.

## Figures and Tables

**Table 1:** SLE data (K) obtained by DSC and POM.

Cholesterol + Phytosterol			Cholesterol + γ-Oryzanol						
*x*_1_	*T_initial_*	*T_fus_*	*x*_1_	*T*_*tr*1_	*T_initial_*	*T*_*tr*2_	*T*_*tr*3_	*T_fus_*	*T_final_*
0.111	404.95	409.84	0.101	319.98	332.9	352.7	363.1	405.36	442.35
0.197		406.15	0.207	318.03	331.2	355.0	363.1	403.15	439.25
0.300	399.15	404.70	0.299	317.83	324.6	350.1	361.3	401.15	436.35
0.408		403.78	0.398	317.41	328.3	350.1	358.1	394.35	423.15
0.496	399.65	405.30	0.499		322.1	341.6	351.1	375.11	413.15
0.600		408.25	0.597	309.74	321.2	342.6		339.77	408.15
0.698		409.05	0.691		329.8			387.94	398.75
0.790	403.15	409.65	0.812	310.89	330.5	338.2	353.2	402.74	408.15
0.899		410.70	0.900	304.57	332.6	331.4		412.15	

*T_fus_* is melting temperature obtained by DSC, *T*_*tr*1_ is the 1st transition observed in DSC, *T_tr_* are other solid transitions, *T_initial_* and *T_final_* are the initial and final temperature observed in POM.

## Data Availability

Data is contained within the article.

## Supplementary Materials

The following supporting information can be downloaded at: https://www.mdpi.com/article/10.3390/foods11223629/s1, Figure S1. Cholesterol + phytosterol system DSC thermograms (from top to bottom, x = 0.0 to 1.0 cholesterol molar fraction); Figure S2. Cholesterol + γ-oryzanol system DSC thermograms (from top to bottom, x = 0.0 to 1.0 cholesterol molar fraction).
