# Supplementary material for: Phytosterols and γ-Oryzanol as Cholesterol Solid Phase Modifiers during Digestion
Source: Foods. 2022 Nov 14;11(22):3629. doi: 10.3390/foods11223629 (PMC9689245; doi:10.3390/foods11223629)
Supplement: Supplementary file 1 [file foods-11-03629-s001.zip › foods-1939148-supplementary.pdf]

Supplementary Material

Phytosterols and γ-oryzanol as cholesterol solid phase modifiers during digestion

Eduardo S. Esperança,[a] Mariane S. Bonatto,[a] Karen C. G. Silva,[a] Gustavo G. Shimamoto,[b] Matthieu Tubino,[b] Mariana C. Costa,[c] Christianne E. C. Rodrigues[d], Antonio J. A. Meirelles,[a] Ana C. K. Sato,[a] and Guilherme J. Maximo*[a]

[a] School of Food Engineering, University of Campinas, Campinas, Brazil; [b]. Chemical Institute, University of Campinas, Campinas, Brazil; [c] School of Chemical Engineering, University of Campinas, Campinas, Brazil; [d] Faculty of Animal Science and Food Engineering, University of São Paulo, Pirassununga, Brazil. *maximo@unicamp.br

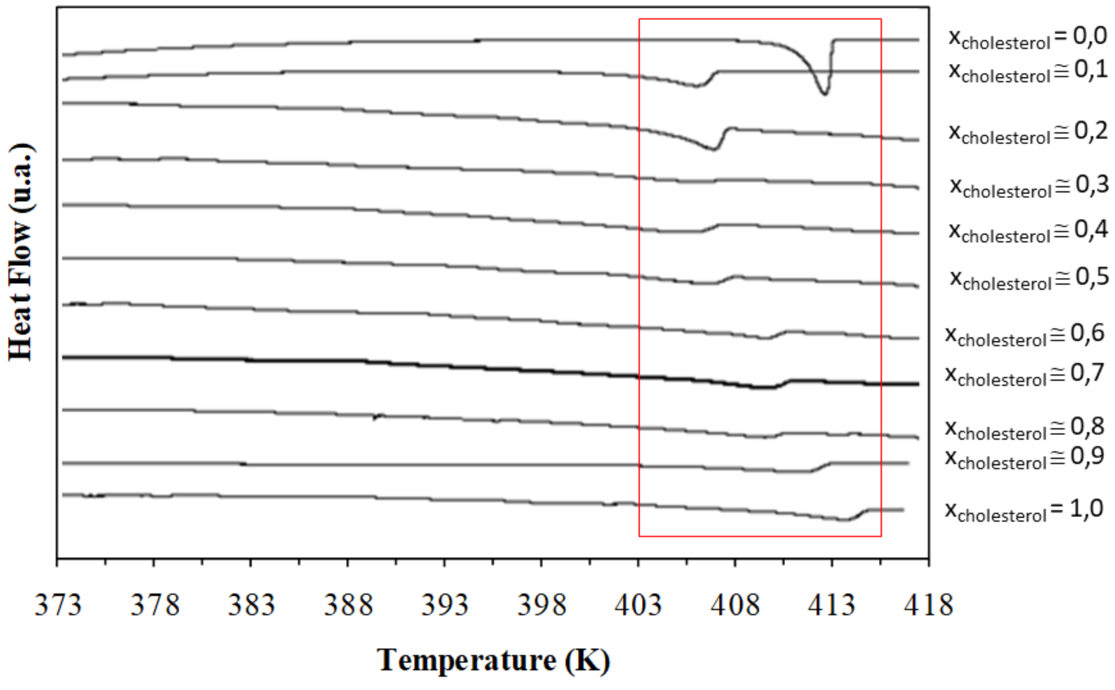

Fig. S1. Cholesterol + phytosterol system DSC thermograms. The red frame highlights where peak top temperatures were obtained.

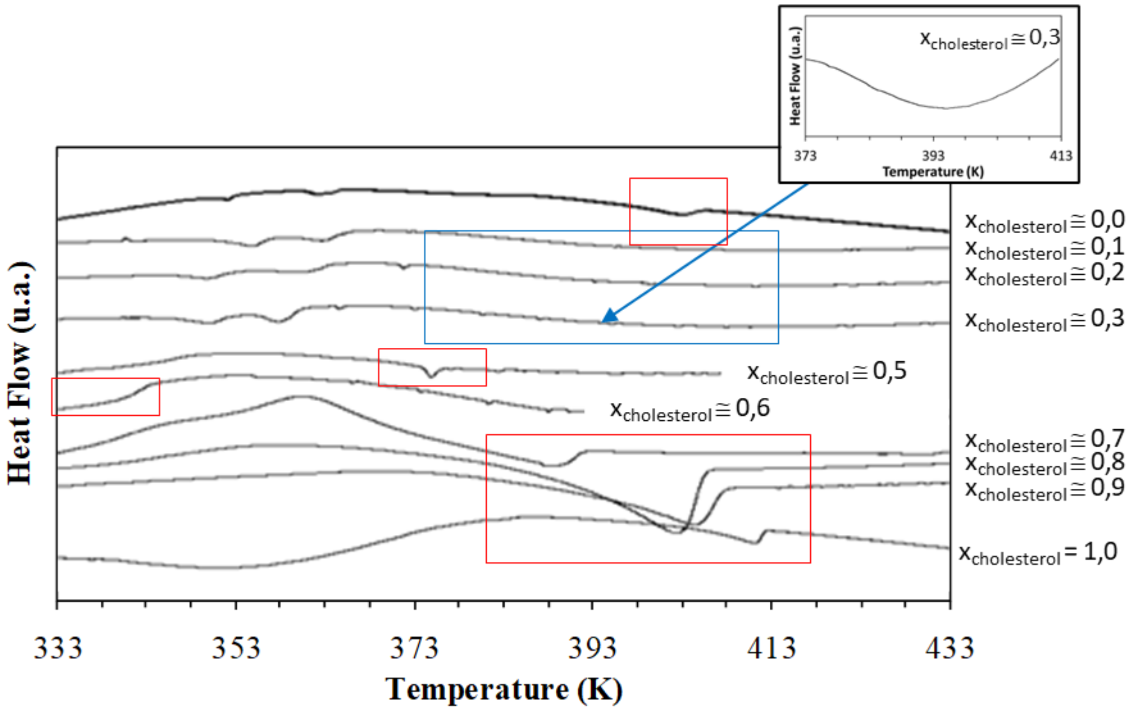

Fig. S2. Cholesterol + γ-oryzanol system DSC thermograms. Blue frame indicates a region where peaks are not intense, and magnification was applied to obtain temperatures. In detail, an example of the magnification showing the mixture $x \sim 0.3$ and the peak top temperature. Red frames highlight regions where other peak top temperatures were obtained.
